# Healthcare workers’ views on the use of continuous positive airway pressure (CPAP) in neonates: a qualitative study in Andhra Pradesh, India

**DOI:** 10.1186/s12887-018-1311-8

**Published:** 2018-11-06

**Authors:** Juan Emmanuel Dewez, Harish Chellani, Sushma Nangia, Katrin Metsis, Helen Smith, Matthews Mathai, Nynke van den Broek

**Affiliations:** 1 Centre for Maternal and Newborn Health, Liverpool School of Tropical Medicine, Pembroke Place, Liverpool, L3 5QA UK; 2 Department of Paediatrics, Vardhman Mahavir Medical College & Safdarjung Hospital, Ring Road, Safdarjung West, Safdarjung Campus, Ansari Nagar East, New Delhi, Delhi, 110029 India; 3 Department of Neonatology, Lady Hardinge Medical College & Kalawati Saran Children’s Hospital, C-604, Shaheed Bhagat Singh Road, Diz Area, Connaught Place, New Delhi, Delhi, 110001 India

**Keywords:** CPAP, Non-invasive ventilation, Neonatal care, Quality of care, India, Qualitative research

## Abstract

**Background:**

Continuous Positive Airway Pressure (CPAP) is a form of non-invasive ventilatory support which is increasingly used in low- and middle-income countries to treat neonates with acute respiratory distress. However, it may be harmful if used incorrectly. We aimed to explore the experiences of doctors and nurses using CPAP in neonatal units in India and their views on enablers and barriers to implementation of CPAP.

**Methods:**

Participants from 15 neonatal units across Andhra Pradesh were identified through purposive sampling. Eighteen in-depth interviews (IDI) with doctors and eight focus group discussions (FGD) with 51 nurses were conducted. Data were analysed thematically using the framework approach.

**Results:**

Common structural factors that limit the use of CPAP include shortages of staff, consumables and equipment, and problems with regard to the organisation of neonatal units in both district hospitals and medical colleges. This meant that CPAP was often not available for babies who were identified to need CPAP, or that CPAP use was not perceived to be of the highest quality. Providing care under constrained circumstances left staff feeling powerless to provide good quality care for neonates with acute respiratory distress. Despite this, staff were enthusiastic about the use of CPAP and its potential to save lives. CPAP use was mostly perceived as technically easier to provide than ventilation and allowed nurses to provide advanced neonatal care, independently of doctors.

**Conclusions:**

Doctors and nurses embraced CPAP use but identified barriers to implementation which will need to be addressed in order not to impact on safety and quality of care. Ensuring a supportive and enabling environment is in place will be crucial if CPAP is to be scaled-up more widely.

**Electronic supplementary material:**

The online version of this article (10.1186/s12887-018-1311-8) contains supplementary material, which is available to authorized users.

## Background

An estimated 2·9 million neonates die every year worldwide. Prematurity, intra-partum related conditions, and infections together account for the majority of all neonatal deaths [1]. Acute respiratory distress is common to these causes of death and is associated with case fatality rates as high as 20% in low- and middle-income countries (LMICs) [2]. Respiratory support to manage acute respiratory distress in neonates can be provided by continuous positive airway pressure (CPAP) or mechanical ventilation [3]. Mechanical ventilation entails endotracheal intubation, an invasive procedure requiring advanced technical skills. CPAP is a non-invasive form of respiratory support which does not require complex technical expertise. Moreover, simple CPAP devices, such as bubble CPAP, which are easier to maintain and repair, have been developed recently [2]. For these reasons, there is growing interest to scale up the use of CPAP in neonates with acute respiratory distress in LMIC settings [4–8].

The World Health Organization (WHO) recommends the use of CPAP for the treatment of preterm infants with respiratory distress [9]. However, the use of CPAP in neonates may lead to rare, but potentially serious complications, such as pneumothorax, nasal trauma, retinopathy of prematurity (ROP), and sepsis [10–13]. The availability of structures and processes required to safely deliver CPAP, must be considered prior to its introduction and scale-up in LMIC settings [9].

In India, where 779,000 neonatal deaths occur every year [1], CPAP is implemented in 68% of government medical colleges and 36% of government district hospitals. However, some hospitals in India lack some of the ancillary infrastructure needed to use CPAP (e.g. radiography, or new respiratory circuits for each patient) (Dewez JE, Nangia S, Mathai M, Chellani H, White S, Francis P et al. The availability and quality of continuous positive airway pressure (CPAP) for neonates in public health facilities in India: a cross sectional survey, submitted), which may discourage healthcare workers to use it. Little is known about the views of doctors and nurses about using CPAP in neonates in hospitals with limited resources. These views are essential to inform future scaling-up of this intervention in India and other LMIC settings.

The aim of this study was to explore the views of healthcare workers regarding the use of CPAP in neonates in the working environment of a middle-income country, and provide suggestions for improvement, if needed.

## Methods

### Objectives, design and setting

The study had three objectives: 1) to explore the perspectives and experiences of healthcare workers on the use of CPAP in neonates in a middle-income country: 2) to identify what might help or hinder implementation of CPAP; and 3) to provide suggestions for what would be needed to improve the use of CPAP and further scale up in India and other middle-income countries.

To achieve these objectives, a qualitative study was conducted with healthcare workers from hospitals in Andhra Pradesh, India. Andhra Pradesh located in south east India, is the eighth largest state in India, covering an area of 160,205 km and with a population of 53 million [14]. The institutional delivery rate was 91.5% in 2015 (of which 38.3% were in government facilities), compared to the Indian average of 83% [15, 16]. The newborn mortality rate in 2015 was 28 /1000 live births, in line with the Indian average [16].

CPAP was introduced in neonatal units of government facilities in Andhra Pradesh from 2007, but most facilities started using CPAP from 2012 onwards (see Additional file 1 for details). The Indian Ministry of Health and Family Welfare recommends that CPAP should be used in tertiary centres (medical colleges) only. However, CPAP has also been introduced in level 2 facilities (district hospitals) in Andhra Pradesh following local initiatives, such as the donation of CPAP machines by wealthy entrepreneurs, and through the support of UNICEF within its broader programme of supporting the implementation of Special Newborn Care Units. UNICEF, in partnership with the state health authorities, developed clinical guidelines, organised workshops to train health care workers, and supportive supervision in hospitals to assist with the implementation of CPAP.

### Sampling and recruitment

We used purposive sampling. We included two types of hospitals (district hospital, and medical colleges) and two types of healthcare workers (doctor and nurse) to ensure enough diversity of views. The inclusion criteria for hospitals were that they had to be from the government sector and that their neonatal unit(s) had to be equipped with CPAP machines (at least one conventional bubble CPAP machine). Moreover, we included hospitals from the north and south of Andhra Pradesh, and ensured the sample included district hospitals and medical colleges. We developed a sampling frame by contacting all neonatal units of Andhra Pradesh. Of the 19 hospitals identified as using CPAP, 15 healthcare facilities (eight district hospitals and seven medical colleges; seven hospitals from the north and eight hospitals from the south) were selected (see Additional file 1 for hospital characteristics). The inclusion criteria for healthcare workers were that they had to be working in the neonatal unit of the selected hospitals, clinically active, and providing CPAP care to their patients.

Focus group discussions (FGDs) were conducted with nurses to elicit norms and group-shared experiences of using CPAP in their neonatal units. Doctors were not available in sufficient numbers for FGDs without significantly disrupting clinical activities, so in-depth interviews (IDI) were conducted with this cadre.

It was estimated that eight focus group discussions (FGDs) and 20 in-depth interviews (IDIs) would be sufficient to reach data saturation (Table 1), based on sample sizes usually used in qualitative research [17], and on discussions within the team about how much variability of views on the topic of interest there may be.

**Table 1:** Participants in in-depth interviews (IDIs) and focus group discussions (FGDs) from neonatal units in Andhra Pradesh

District hospitals (secondary level of care)					Medical colleges (tertiary level of care)				
Neonatal unit	Doctors (IDIs)		Nurses (FGDs)		Neonatal unit	Doctors (IDIs)		Nurses (FGDs)	
	A	B	A	B		A	B	A	B
Unit 1	1–2	1	6–8	7	Unit 1	1–2	1	6–8	7
Unit 2	1–2	1	6–8	6	Unit 2	1–2	1	6–8	6
Unit 3	1–2	1	6–8	7	Unit 3	1–2	1	6–8	6
Unit 4	1–2	1	6–8	6	Unit 4	1–2	1	6–8	6
Unit 5	1–2	1	–		Unit 5	1–2	1	–	
Unit 6	1–2	1	–		Unit 6	1–2	2	–	
Unit 7	1–2	1	–		Unit 7	1–2	2	–	
Unit 8	1–2	2	–						
Total	8–16	9	24–32	26		7–14	9	24–32	25

A Sampling framework, B Number of recruited participants

The research team visited all hospitals included in the study. The time of the visit was agreed in advance with the Head of Department. On the day of the visit, one of the doctors working in the neonatal care unit was invited to take part in an IDI. All doctors agreed to participate in the study. Prior to the visit, the Head of Departments invited at least six nurses to take part in an FGD. We do not know if some nurses declined to participate as we did not request this information.

### Data collection

Data collection took place between May and August 2016. Data were collected by two local female nurses experienced in social research and by a male paediatrician (JED) trained in qualitative research. JED’s research area of interest is the feasibility of using new technologies for child care in LMICs. Interviews were conducted in the participants’ health facilities. No one other than researchers and participants were present.

A topic guide was developed for FGDs and IDIs. The topic guide for FGDs was translated from English to Telugu, the local language of Andhra Pradesh, by the two local nurses who were fluent in both English and Telugu. The nurses conducted the FGDs in Telugu. JED was present during the FGDs and a debriefing was organised at the end of each FGD to discuss whether the topic guide needed to be amended, in light of new topics arising from the FGDs. The debriefings and the different amendments made iteratively to the topic guide jointly by JED and the two nurses ensured that the latter had a good understanding of the objectives of the study and that the topic guide was used in the best way to address the research questions, and consistently throughout the FGDs. The topic guides were used to ensure important aspects were covered but were used flexibly so participants could elaborate on other points that were important to them. The topic guide (Additional file 2) included the following domains: the experience of the healthcare provider, the perceived benefits and potential limitations of CPAP as an intervention to treat newborns with respiratory distress, the different aspects of CPAP and factors promoting or hindering the application of these, potential complications of CPAP, the type of support healthcare providers required to be able to use CPAP. All FGDs were audio-recorded, fully transcribed and translated from Telugu to English by one of the nurses who conducted the FGDs. All IDIs were conducted in English by JED, as all included doctors were fluent in English. IDIs were audio-recorded, and fully transcribed by JEDs.

No formal recommendation about corrective measures to address potential inappropriate use of CPAP was provided to participants. To avoid any concerns among participants that malpractice would be reported, we clarified in the consent process that clinical practice was not being assessed, and that recommendations arising from the study would be for all healthcare facilities in general and not for individual facilities. However, the research team provided informal clarifications at the end of the IDIs and FGDs when participants made specific requests.

### Data analysis

The thematic framework approach, a transparent matrix-based approach suited to applied research, was used to analyse the data [18]. The analysis was mainly inductive in nature. An initial list of codes was generated through line-by-line coding of a selection of the transcripts of the IDIs and FGDs (Additional file 3). Transcripts were read by a second researcher who independently generated another set of initial codes. Both researchers then agreed on an analytical framework comprising the most relevant codes, which was used to manually code the entire data set. Using matrices to display data relevant to each theme, further exploration and interrogation of the data identified similarities and differences across the dataset (Additional file 4). Through discussions with the whole research team, themes were further refined to provide clear descriptions and explanations. Five final themes were identified that reflect participants’ perspectives and experiences, and, relate to the use of CPAP in neonates.

## Results

Eighteen IDIs with male (*n* = 11) and female (*n* = 7) doctors were conducted in the 15 hospitals (Table 2). The sample included nine junior doctors and nine senior doctors. The eight FGDs involved 51 nurses from 8 of the study hospitals. Most of the nurses (*n* = 36) had more than five years’ experience in providing neonatal care.

**Table 2:** Background of participants

	District hospitals (secondary level of care)	Medical colleges (tertiary level of care)	Total
Doctors (7 females, 11 males)			
Junior doctors (paediatric trainees or medical officers)	6 (2 males, 4 females)	3 (all female)	9
Senior doctors (consultant paediatricians or consultant neonatologists)	3 (all male)	6 (all male)	9
Total doctors	9	9	18
Nurses (all female)			
< 5 years of experience in neonatal care	24	12	36
> 5 years of experience in neonatal care	2	13	15
Total nurses	26	25	51

The five main themes which emerged included: 1) Shortages of supplies, infrastructure, and staff mean CPAP is not always available and/or of the highest quality; 2) Poor organisational support hinders optimal implementation of CPAP and neonatal care; 3) Healthcare providers feel powerless to provide better care for neonates; 4) Healthcare providers perceive CPAP as a beneficial intervention; and 5) CPAP enables nurses to work independently. These are described below with illustrative quotes for each theme provided in Table 3.

**Table 3:** Illustrative quotes from identified themes

Theme 1: Shortages of supplies, infrastructure, and staff mean CPAP is not always available and/or of the highest quality	
“With that budget we can use, say 10 circuits (per 3 months), whereas we have 6 new cases who need CPAP per week. So, we are obliged to reuse circuits, humidifiers, and prongs. These are not autoclavable, we disinfect them but this is not ideal.” *(Consultant Neonatologist, medical college, IDI 6)*	
“Equipment is not available, I mean the sizes of the prongs, sufficient caps and all that equipment are not available. We haven’t been able to use it (CPAP) as much as we should.” *(Medical Officer, medical college, IDI 17)*	
“We only have three CPAP machines. Sometimes more than five babies need CPAP at the same time” *(Nurse, medical college, FGD 1)*	
“We have 20 CPAP machines, but only five or six are working. If we get more preterm babies, we have a shortage.” *(Senior Resident, medical college, IDI 18)*	
“The main problem is the number of babies compared to the number of personnel. At times, we are not able to deal with cases needing urgent care. Sometimes we have three babies in a serious condition and only one doctor to take care of them.” *(Senior Resident, medical college, IDI 1)*	
“Our main issue is shortage of staff in nursing. We are sometimes rotated to other wards and we face emergencies especially in the evenings and nights. We may have to deal with 20–30 babies and there will be just the two of us. Feeding has to be done at almost the same time. How can two of us do it?” *(Nurse, medical college, FGD 6)*	
“We put him under CPAP, he improved but then we had a power cut which lasted for many hours, 6 h, and he immediately deteriorated. We had to intubate him to refer him but the vehicle for transfer took too much time to come. Then he died before we could refer him” *(Medical Officer, district hospital, IDI 4)*	
“I don’t think we had a case of pneumothorax, but as I mentioned we don’t do x-rays as we had no mobile X-ray machine.” *(Doctor, district hospital, IDI 4)*	
“Because of lack of monitoring, some babies go into (cardiac) arrest or apnoea, and nobody is there, if apnoea is not corrected they go into arrest and babies die immediately, within a fraction of seconds. So, if more staff was there, babies could be monitored better.” *(Senior Resident, medical college, IDI 17)*	
“Because there is a rush of cases and the shortage of staff, and the fact that CPAP requires continuous monitoring, we are not able to focus on non-CPAP cases.” *(Nurse, medical college, FGD 1)*	
Theme 2: Poor organisation support hinders optimal implementation of CPAP and neonatal care	
“If they were guidelines on how to use the money, then the Head of Department and the supplier would communicate better. We need a better process of stock management and supply delivery.” *(Consultant Neonatologist, medical college, IDI 6)*	
“Some of the nurses rotate after 3–6 months to other wards (internal medicine, psychiatry), and so, in this short period of time they do not gain enough confidence to enjoy using this device (CPAP).” *(Senior Resident, medical college, IDI 3)*	
“I was alone one day in the ward and there was a power shut down for nearly 5–6 h. There was a baby on CPAP. I began giving oxygen through ambu (bag and mask). There was a security guard outside. He was helping me with other things, but to ventilate with ambu, you need to be trained technically. I asked my superiors for help, to send my colleagues (who were in OT or labour room) or any other nurses to assist me. They told me to manage with the security guard. When I told them that he does not know how to use the ambu, they told me that I should have anticipated this situation and should have trained him in preparation.” *(Nurse, district hospital, FGD 5)*	
“Whenever we get into a crisis because of a baby dying, no one comes to support us. If it happens, the administrators tell us that they cannot be responsible for what we have done. There is absolutely no support. Everyone treats the newborn unit as an external unit.” *(Nurse, district hospital, FGD 5)*	
“We don’t know who will solve our problems, whoever we ask, they blame each other, they say they are not responsible as we are under the jurisdiction of the central government.” *(Nurse, medical college, FGD 6)*	
“The entire hospital does not value our services. By this I mean that nobody supports us. Not even the nursing superintendent understand us. We feel isolated. They say since we are from the National Health Mission and they are from the state government, we have to go to the National Health Mission to deal with our problems. There is no one to represent us in the hospital.” *(Nurse, medical college, FGD 1)*	
Theme 3: Healthcare providers feel powerless to provide better care for neonates’	
“The power in our hospital comes and goes every 5–15 min. At times, we run out of oxygen cylinders and we have to rush to place an indent and order it. Sometimes, the cylinders are not available even in the store. We become so helpless. We lose our confidence, especially when we are alone.” *(Nurse, district hospital, FGD 5)*	
“The night can be quite hectic, because if three emergency cases are admitted, at least one nurse should be with the doctor to resuscitate the baby, otherwise how can we do it? how can the sisters cope?” *(Medical Officer, district hospital, IDI 7)*	
“The work load is very high and in the afternoon, we are tired and we are not able to offer 100% of the care. In the long term this may have an impact on our motivation.” *(Consultant Paediatrician, medical college, IDI 2)*	
“Sometimes we are alone. The rest of us must go to OT and labour ward. There is so much pressure to give EBM, monitor vitals, handle admissions, sometimes deal with oxygen shortage, giving ambu, connect to ventilator, etc. It is so much stress.” *(Nurse, district hospital, FGD 5)*	
“When the power goes off, there is no air flowing. We ourselves feel frustrated. We can only imagine how the babies must be feeling.” *(Nurse, district hospital, FGD 7)*	
“Yes. Everything of CPAP, we reuse: nasal prongs, circuits, caps. We do not have the choice (…) We are happy to see the baby is given CPAP but also fear and pray that there should not be any infections because of reusing the prongs.” *(Nurse, district hospital, FGD 7)*	
Theme 4: Healthcare providers perceive CPAP as a beneficial intervention	
“We are still using CPAP (despite all the constraints), we don’t want to lose the babies” *(Consultant Neonatologist, medical college, IDI 13)*	
“Definitely, I would recommend it, it (CPAP) has a positive impact on preterm and term babies, but still we have to improve the quality of our work” *(Senior Resident, medical college, IDI 18)*	
“There are more advantages than disadvantages of using CPAP” *(Nurse, district hospital, FGD 7)*	
“It prevents the need for mechanical ventilation, prevents the complications of mechanical ventilation, the comfort of the baby is better” *(Medical Officer, district hospital, IDI 9)*	
“Earlier, when we got babies with severe respiratory distress, we used to refer them to other facilities. Now we can manage it ourselves.” *(Nurse, district hospital, FGD 2)*	
“CPAP is an easy procedure, we can use it here in a safe way” *(Medical Officer, district hospital, IDI 10)*	
Theme 5: CPAP enables nurses to work independently	
“It is easy to place, easy to remove; it is not difficult to use CPAP.” *(Nurse, medical college, FGD 6)*	
“We have no problem in connecting the CPAP and we are well trained and are able to operate the machine with ease.” *(Nurse, district hospital, FGD 7)*	
“Using CPAP is very simple and [nurses] can do it independently, they don’t need to ask us, whereas the monitoring of mechanical ventilation needs a lot of technical expertise. With CPAP, [nurses] can take their own decisions and act very well.” *(Senior Resident, medical college, IDI 3)*	
“We do not need doctors to operate CPAP. Doctors are very busy and mostly not available. We can operate the CPAP by ourselves. We cannot do the same with ventilators” *(Nurse, medical college, FGD 1)*	
“We, nurses, operate the machines on our own, many times without the help of doctors- be it identifying the baby who requires CPAP or placing the prongs, positioning the baby, or recording the Silvermann score. We do it by ourselves” *(Nurse, medical college, FGD 8)*	

### Shortages of supplies, infrastructure, and staff mean CPAP is not always available and/or of the highest quality

Shortages of consumables and equipment and problems with infrastructure were common problems and had been experienced in all settings. Most participants reported shortages of specific consumables such as nasal prongs and respiratory circuits. Masks and equipment were sometimes available but in sizes ‘that are not suitable’ for preterm babies. Doctors from only two healthcare facilities reported that consumables were in good supply. Some doctors and nurses argued that additional CPAP machines were needed, because there were ‘not enough machines’ or many machines were broken. Although maintenance services were generally available, the service provided was often described as “not timely” and “not always effective”.

Shortages of staff was also a common problem reported in all interviews. Participants reported frequent ‘rotation’ of staff. When nurses trained in neonatal care were partially deployed in other wards, they lost ‘confidence’ and skills in the use of CPAP effectively. This was reported more in district hospitals than in medical colleges.

Most doctors and nurses had found it difficult to provide respiratory support with CPAP when they were facing these constraints, which were perceived as ‘compromising’ the care provided. Some doctors and nurses reported they could only provide standard care (oxygen and antibiotics), or had to use mechanical ventilation (which they considered as potentially harmful) when consumables and equipment for CPAP were insufficient. Others reported disinfecting and re-using consumables for CPAP, or modifying nasal prongs of inappropriate size, which they recognised was ‘not ideal’. The lack of a functioning X-ray machine was reported as problematic by some doctors because this meant pneumothorax could not be diagnosed. Half of the nurses reported that they had to sometimes (reluctantly) wean babies off CPAP too early to provide CPAP to newly admitted sick neonates. Staff from medical colleges had a higher caseload and were, overall, more concerned and had faced more of these problems compared to healthcare providers from district hospitals.

### Poor organisational support hinders optimal implementation of CPAP and neonatal care

The way in which neonatal units are organised and run was reported by healthcare providers as being the cause of many of the constraints faced. Some doctors and most nurses believed they were not supported by the hospital management team or local authorities. In many cases, doctors and nurses described how their neonatal units were regarded as ‘external’ to the rest of the hospital or as distinct structures because they were under the authority of the National Health Mission, a federal government initiative aimed at improving neonatal health. The co-existence of two parallel authorities (the hospital management and the National Health Mission) created confusion about which authority was responsible for staffing and supplies, leading to major supply shortages and redeployment of staff reported above.

### Healthcare providers feel powerless to provide better care for neonates

The struggle to provide care under ‘quite hectic’ circumstances and sometimes being ‘overwhelmed’ by patient numbers affected staff and made one doctor question ‘how can the sisters cope?’. There was a sense of ‘powerlessness’ expressed by most nurses and some doctors when faced with shortages of the basic supplies required for CPAP as well as by electricity “blackouts”. Some participants, particularly doctors, felt they were ‘overwhelmed’ by the number of cases requiring respiratory support. Working conditions were perceived as ‘stressful’ by some nurses, others described how they were ‘struggling’. Some nurses felt guilty for not being able to provide better care. Some nurses were afraid of harming neonates because of the need to reuse consumables.

### Healthcare providers perceive CPAP as a beneficial intervention

Despite the constraints, almost all doctors and nurses mentioned that they ‘are still using CPAP’. Most doctors said that they would recommend it to colleagues from other healthcare facilities. The benefits of using CPAP in terms of preventing the ‘need for’ and the ‘complications of’ mechanical ventilation were recognised by most of the healthcare providers. Doctors and nurses highlighted that CPAP allowed efficient respiratory support for small neonates and reduced the use of mechanical ventilation. Moreover, not having to refer babies to a better equipped healthcare facility was seen as a major benefit of CPAP. Many doctors and nurses mentioned that fewer “referrals out” had been needed since they commenced using CPAP, and that they were better able to manage babies with respiratory distress in general. Organising referrals was difficult given the scarcity of ambulances, the need to convince parents to move to another distant facility, and because neonates were at risk of deteriorating during the transfer. Doctors reported that they had noticed a reduction in neonatal mortality in their units which they attributed to the use of CPAP. In terms of complications, all nurses and almost all doctors reported that they saw few complications when using CPAP, and that any harm caused to neonates was mainly related to the different constraints described above, rather than to CPAP itself.

### CPAP enables nurses to work independently

Almost all doctors and nurses referred to CPAP as an ‘easy procedure’ or that they could ‘operate the machine with ease’. Because of the perceived technical simplicity of applying CPAP, most nurses and some doctors felt that trained nurses could initiate CPAP ‘independently’. This is in sharp contrast to mechanical ventilation which required doctors to be present. Nurses were confident and felt satisfied that they ‘do not need doctors to operate CPAP’ and that ‘we nurses operate the machines on our own…without the help of doctors’. Allowing nurses to be more independent in providing advanced care to neonates was reported to, at least partially, resolve the bottleneck caused by lack of availability of doctors.

## Discussion

### Main findings

To the best of our knowledge, this is the first study to explore the perceptions and experiences of healthcare workers from a middle-income setting about their use of CPAP in neonates, and the factors affecting implementation of CPAP in newborn care units in such settings. Healthcare providers working in neonatal units in this study faced several constraints that hindered the implementation of CPAP. Shortages of staff, equipment, consumables and a non-supportive work environment meant that healthcare providers could not always provide CPAP when they knew it was needed for a baby, nor could they provide the level and quality of care they would have liked to. Providing care under these circumstances can leave staff feeling “powerless”. But despite such constraints, healthcare providers recognised the potential of CPAP as a life-saving treatment. Using CPAP was perceived as technically straightforward and it allowed nurses to provide advanced care to neonates “independently of doctors”, which is particularly helpful given the shortage of other trained specialist staff at neonatal units in district and tertiary level healthcare facilities.

Staff shortages were explained by the feeling that the management teams in government hospitals tend to not perceive neonatal care units as akin to intensive care units in terms of staffing requirements but consider that the nurse-patient ratio can be the same as for the general paediatric wards. Other studies in low and middle-income settings have identified similar situations, where staff report that there are too few healthcare workers in place to manage babies who require CPAP and to ensure suitable regular monitoring is provided [19]. The global Every Newborn Action Plan to reduce neonatal mortality, emphasises that availability of human resources and sustainable supply chains for medical products are major bottlenecks to the provision of quality inpatient neonatal care [20]. The Indian Newborn Action Plan also recognises that quality of care in hospitals is affected by a lack of human resources [21]. The findings of this study further highlight the need to address staff, as well as consumables and equipment shortages when new care packages such as CPAP are introduced and/or scaled up in a country.

Healthcare providers reported that there were problems regarding the organisational structure related to recruitment, management, and supply of consumables and equipment. Most neonatal units in India have been established by the federal government under the National Health Mission but are located within hospitals under the jurisdiction of state governments. Because the responsibilities of both authorities in this setting are unclear to many healthcare providers and managers, there can be problems with allocation of budget, human resources, consumables and equipment. The World Health Organization recommends that every health facility should have enough managerial and clinical leadership to foster an enabling environment, including a supportive environment for the staff, to improve the quality of newborn care [22]. Our findings provide an illustration of the impact on working conditions of the lack of leadership and organisational capabilities in health service.

The fact that healthcare workers recognised CPAP as a new evidence-based treatment option for babies with neonatal distress and are enthusiastic about using CPAP was surprising but similar findings were reported from South Africa [23]. Moreover, in terms of CPAP allowing nurses to be more independent, studies from Fiji and Ghana also reported that nurses could competently administer CPAP in the absence of doctors [6, 19].

### Limitations of the study

This study includes the perspectives of both doctors and nurses using CPAP in neonates. A female nurse fluent in the local language and experienced in interviewing healthcare workers facilitated the FGDs with nurses to obtain rich and detailed information. However, it was not possible to interview hospital managers, state health authorities, nor maintenance technicians, whose perspectives would also be valid and would add another dimension to the data, because of time and resources constraints. Purposive sampling was used to recruit a range of junior and senior doctors, nurses with different level of experience, and staff from two levels of care. However, nurses were selected by the Head of Department, which may have introduced selection bias. Finally, this study only sought to explore the experiences and perceptions of healthcare workers about the use of CPAP, and not to assess their knowledge and skills, which may also impact on optimal use of CPAP.

### Implications for practice and further research

India has shown a strong commitment to reducing neonatal mortality over the last decades. Availability of advanced neonatal care services has increased through major investments by the government; 602 special newborn care units have been established and more will be implemented in the future [24]. However, there are also concerns about the quality of care provided in these units. This study illustrates what is needed to introduce and scale-up an additional care package such as CPAP care in this context. Availability and quality of CPAP rely not only on “the machine” being there but on a complete enabling environment including trained staff, consumables, maintenance, and ability to diagnose and manage complications of CPAP use in neonates. Staff motivation to use new technologies is high. But, policy makers and managers should be aware that staff motivation could be adversely affected in the long term if constraints in the working environment are not addressed satisfactorily. These include staff training and a better staff-patient ratio. Efforts should also be put in place to improve the integration of neonatal units with the rest of the hospitals, better organization particularly in terms of providing regular supplies of consumables, and strengthening accountability through monitoring and supportive supervision. Operational research should investigate the quality of CPAP use, particularly the knowledge and skills of the staff using CPAP, and the impact of the introduction and scale-up of CPAP on important clinical outcomes such as neonatal mortality, and adverse events.

## Conclusion

Healthcare providers working in neonatal units in Andhra Pradesh report constraints when using CPAP, particularly shortages in staff, consumables and equipment. These constraints impact on the optimal use of CPAP in neonatal units. Despite these constraints, healthcare providers understand and have experienced the benefits of CPAP and want to use CPAP in neonates whenever possible and required. Using CPAP is perceived to be easy to use in comparison with mechanical ventilation, even though it requires considerable resources and expertise, and that it allows nurses to be more independent. These findings can be used to encourage the use of CPAP in other countries moving towards advanced neonatal care. However, policy makers and managers will need to address the problems identified in this study before planning further scale up of the use of CPAP. Further research is needed to assess whether the use of CPAP in the context of the constraints described in this study leads to an improvement of neonatal survival and health in middle income countries.

## Additional files

Additional file 1: Characteristics of included hospitals. Provides information on level of care provided and workload relevant to CPAP use. (DOCX 13 kb)

Additional file 2: Topic Guide. Provides the topic guide used in the study. (DOCX 14 kb)

Additional file 3: Initial analytic framework. Describes the framework used for data analysis. (DOCX 15 kb)

Additional file 4: Example of a summary matrix. Shows the summary matrix for an emergent theme “CPAP as a beneficial intervention”. (DOCX 23 kb)

## Abbreviations

CPAP: Continuous positive airway pressure
FGD: Focus group discussion
IDI: In-depth interviews
LMIC: Low- and middle-income countries
MIC: Middle-income countries
ROP: Retinopathy of prematurity
