# Supplementary material for: Healthcare workers’ views on the use of continuous positive airway pressure (CPAP) in neonates: a qualitative study in Andhra Pradesh, India
Source: BMC Pediatr. 2018 Nov 6;18:347. doi: 10.1186/s12887-018-1311-8 (PMC6220518; doi:10.1186/s12887-018-1311-8)
Supplement: Supplementary file 1 — Characteristics of included hospitals. Provides information on level of care provided and workload relevant to CPAP use. (DOCX 13 kb) [file 12887_2018_1311_MOESM1_ESM.docx]

**Additional file 1: Characteristics of included hospitals**

| **Hospital** | **Level of care** | **Location in Andhra Pradesh** | **Average monthly Neonatal admissions** | **Beds** | **Average number of patients monitored by one nurse** | **Year of introduction of CPAP** | **Average proportion of admissions who receive CPAP (%)** |
| --- | --- | --- | --- | --- | --- | --- | --- |
| **1** | Level 3 | North | 128 | 50 | 13 | 2007 | 16 |
| **2** | Level 2 | North | 137 | 35 | 9 | 2016 | 1 |
| **3** | Level 3 | North | 274 | 50 | 17 | 2012 | 10 |
| **4** | Level 2 | North | 93 | 24 | 6 | 2013 | NA |
| **5** | Level 2 | North | 126 | 32 | 4 | 2013 | 3 |
| **6** | Level 3 | North | 279 | 110 | 28 | 2013 | 5 |
| **7** | Level 2 | North | 128 | 22 | 11 | NA | 1 |
| **8** | Level 3 | South | 175 | 60 | 15 | 2016 | 8 |
| **9** | Level 2 | South | 92 | 25 | 4 | 2016 | 1 |
| **10** | Level 2 | South | 85 | 15 | 3 | 2016 | 1 |
| **11** | Level 3 | South | 163 | 50 | 8 | 2013 | 14 |
| **12** | Level 2 | South | 76 | 20 | 4 | 2014 | 2 |
| **13** | Level 3 | South | 206 | 21 | 7 | 2015 | 17 |
| **14** | Level 2 | South | 128 | 25 | 6 | 2013 | 6 |
| **15** | Level 3 | South | 262 | NA | NA | 2012 | 31 |
