# Supplementary material for: Healthcare workers’ views on the use of continuous positive airway pressure (CPAP) in neonates: a qualitative study in Andhra Pradesh, India
Source: BMC Pediatr. 2018 Nov 6;18:347. doi: 10.1186/s12887-018-1311-8 (PMC6220518; doi:10.1186/s12887-018-1311-8)
Supplement: Supplementary file 2 — Topic Guide. Provides the topic guide used in the study. (DOCX 14 kb) [file 12887_2018_1311_MOESM2_ESM.docx]

**Additional file 2: Topic guide**

Thank you for participating in the study, we are really interested to know about what you think of using CPAP in your unit,

1. Can you tell us about yourselves, for how long have you been working in the unit, for how long have you been using CPAP?

1. From your experience, what are the benefits, if any, of using CPAP in your unit?
2. What are the drawbacks, if any, of using CPAP in your unit?
3. While some nurses think it is possible to treat newborns effectively with CPAP in newborn units in India, others think it is challenging. What is your opinion of the use of CPAP in your unit?
   1. **If participants mentioned it is possible to provide CPAP effectively in their unit, ask:**
      - 1. Can you elaborate on what factors contribute to this?
   2. **If participants think it is challenging, ask:**
      - 1. Can you elaborate on what factors contribute to this?
        2. What particular aspect of using CPAP in your unit are you concerned about?
        3. What aspect of using CPAP are difficult in your unit?
        4. To what extent do these challenges reduce the use of CPAP by you and your team?
        5. How do you and your team overcome these challenges?
   3. **If participants do not provide sufficient details, ask:**
      - 1. May I ask you to describe the sequence of events in the care provided to a difficult case of newborn requiring CPAP?
        2. What aspects of the care were difficult?
        3. What have you done to overcome these difficulties?
   4. **If participants have not commented on the following aspects, ask explicitly:**

How does your unit manage?

1. Oxygen supply
2. Maintaining a constant pressure
3. Keeping baby calm during CPAP
4. Monitoring of babies
5. Staffing
6. Feeding during CPAP
7. Supply of equipment
8. Availability of technical maintenance
9. Have you experienced any harm to patients because of CPAP use in your unit?
10. If yes, can you describe the harm caused to babies?
11. How is the unit prepared to deal with the harm CPAP may cause to babies?
12. How do parents contribute to the care of babies on CPAP?
13. What other task undertaken by parents would help in the care provided to babies who are under CPAP
14. If the Ministry of Health or an implementer partner approached you offering support for the use of CPAP in your unit, what type of support would you ask for?
15. My final question is to ask each of you what, to you, is the most important thing that has been said, by yourself or anyone else here in the discussion about the use of CPAP in your unit?
16. Is there any other comment you want to add?

Thank you very much for your participation!
