# Supplementary material for: Healthcare workers’ views on the use of continuous positive airway pressure (CPAP) in neonates: a qualitative study in Andhra Pradesh, India
Source: BMC Pediatr. 2018 Nov 6;18:347. doi: 10.1186/s12887-018-1311-8 (PMC6220518; doi:10.1186/s12887-018-1311-8)
Supplement: Supplementary file 3 — Initial analytic framework. Describes the framework used for data analysis. (DOCX 15 kb) [file 12887_2018_1311_MOESM3_ESM.docx]

| **Additional file 3: Initial analytical framework** | |
| --- | --- |
| **Emerging themes** | **Codes** |
| 1. **Positive perception about CPAP use** | - Easiness of using CPAP - Perception about continuation of CPAP - Recommendation of CPAP use - Sense of usefulness - Reduction in referrals - Reduction in MV - Reduction in mortality - Staff expertise and satisfaction - Negative perceptions about using CPAP - Improved Reputation - Positive impact of training |
| 1. **Shortage of supplies** | - Consumables (shortages or no shortages) - General infrastructure (warmers, water, etc) (shortages or no shortages) - Availability of efficient maintenance (availability or lack of) - Consequences of supplies shortages |
| 1. **Shortage of staff** | - Misuse of staff and consequences - Need of Clinical supervision/more specialised paediatric support - Consequences on Workload - Teamwork - Impact on monitoring and general care - Other impact of staff shortage - Lack of training in CPAP use |
| 1. **Parental role** | - Parental access to newborns - Parents not involved in CPAP care - Potential reasons:   - Space issues as a reason   - Not possible to monitor parents   - Hygiene concerns/infection control - Potential consequences |
| 1. **Complication of using CPAP** | - Doubts about harm - Harm and complications (nasal, pneumothorax, sepsis, NEC, ROP, BPD) caused by CPAP - Absence of harm or complications caused by CPAP - Difficulties in identifying complications (no Xray, no ROP screening, no BPD screening) - Difficulties in managing complications (no specific material, no expertise)/preparedness to deal with complications |
