# Supplementary material for: Healthcare workers’ views on the use of continuous positive airway pressure (CPAP) in neonates: a qualitative study in Andhra Pradesh, India
Source: BMC Pediatr. 2018 Nov 6;18:347. doi: 10.1186/s12887-018-1311-8 (PMC6220518; doi:10.1186/s12887-018-1311-8)
Supplement: Supplementary file 4 — Example of a summary matrix. Shows the summary matrix for an emergent theme “CPAP as a beneficial intervention”. (DOCX 23 kb) [file 12887_2018_1311_MOESM4_ESM.docx]

| **Additional file 4. Example of summary matrix for the emergent theme “CPAP as a beneficial intervention”** | | | | | |
| --- | --- | --- | --- | --- | --- |
| **1. Healthcare providers perceive CPAP as a beneficial intervention** | | | | | |
| IDI or FGD | Easiness of using CPAP | Perception about continuation of CPAP/recommendation of CPAP use to other colleagues | Better care to babies (Reduction in treatment failure (death, referrals or use of mechanical ventilation)) | Staff satisfaction (training, expertise, usefulness, improved reputation, independence) | Negative perception about CPAP use |
| IDI 1: SR1SMC level 3 (medical college) | "It is easy (to use CPAP in my facility)" (l16) | "We should continue (despite some difficulties in using it)" (l71) | "The main benefit is to be able to provide better care to preterm babies (l4)" |  |  |
| IDI 2: P1SNCU level 2 (district hospital) |  | "We should continue. If we use CPAP, patients will be better and we will have more time for other aspect of care for other babies." (l142) | "CPAP allows reducing the use antibiotics in those babies with respiratory distress who does not have sepsis but only respiratory problems. Without CPAP we will continue antibiotic because we will have no other choice and we would not know whether the problem is only a respiratory problem." (l91) | "The main factors is the dedication of the staff, the commitment. And also the fact that we like to learn using new technologies" (l145) |  |
| IDI 3: SR2NICU level 3 | "Using CPAP is very simple and they (the nurses) can do it independently, they don’t need to ask us, whereas the monitoring of mechanical ventilation needs a lot of technical expertise and so they ask us. The setting of CPAP are very simple." (l195) | "Yes, as long we are able to manage these complications" (l253)(…) Definitively. I will recommend it because it is a very easy procedure, I would recommend it strongly " (l270) | "CPAP is useful because it decreases the need of mechanical ventilation" (l174) | "They (nurses) can take their own decision and act very well." (l197) |  |
| IDI 4: MO1SNCU level 2 |  | "But I think at least 1 CPAP for a SNCU should be available. It would be beneficial for the patient." (l357) | "It allowed us to treat better a premature baby with respiratory distress, that was the first patient we treated with CPAP and it was successful." (l285) "CPAP will be useful for tachypnoea and RD which are moderately severe. We would be able to manage them here and we would not need to refer them to the capital of the district, as we are doing currently" (l295) "I think it is a great benefit, we can manage more patients and refer less patients." (l352) | "It will be good to use it (again) as work will be more interesting and people will see that we are able to use new technologies and manage moderately severe cases." (l297) |  |
| IDI 5: MO2SNCU level 2 |  | "I would encourage him (a colleague working in another facility), and explain him how we use it here." (l456) | "Since we introduced CPAP, we are referring less patients. The clinical evolution of the babies with RD has improved." (l394) |  |  |
| IDI 6: N1SMC level 3 |  | "Yes, I will definitively advise to use it, and we will offer training." (l539) | "Neonatal Mortality has reduced since we are using CPAP. As a result, the parents’ satisfaction has increased. We are very happy of this development." (l482) | Neonatal Mortality has reduced since we are using CPAP. As a result, the parents’ satisfaction has increased. We are very happy of this development. (l482) | "Nobody has a negative impression of using CPAP." (l484) |
| IDI 7: MO3SNCU level 2 |  | "Even if 2 doctors are on leave, we have to work 12 hours, but it is ok, whenever we can help the baby we will do it. Even if CPAP increases the workload we will use it." (l592) | "We will have less referral for mechanical ventilation. CPAP is an additional intervention which will help us dealing with many of these cases." (l570) | "Another positive is that we can see we are able to deal with this sort of cases, we felt what we did was valuable." (l576) "we felt very bad when we referred cases. Now, we can see we can do something for these babies." (l578) |  |
| IDI 8: MO4SNCU level 3 |  | "I would advise him to do use it." (l836) | "Respiratory distress decreased, mortality decreased,.. improvement of the babies." (l742) | "we have the satisfaction of saving lives." (l744) |  |
| IDI 9: MO5SNCU level 2 |  | "I would recommend it, we can use it in preterm babies, we can even use it with surfactant with the insure technique. There will be less BPD." (l956) | "It prevents the need of mechanical ventilation, prevents the complications of mechanical ventilation, the comfort of the baby is better. Mortality can decrease, we can treat respiratory distress and recurrent apnoea." (l863) |  |  |
| IDI 10: MO6SNCU level 2 | "CPAP is an easy procedure, we can use it here in a safe way." (l987) | "We are saving time. I think it a good thing to use CPAP." (l1009) "I will recommend it." (l1073) | "We can treat respiratory distress, babies are recovering. " (l981) "We have to refer less for mechanical ventilation that is a benefit. We do not have mechanical ventilation here, it may come in the future." (l989) "No, I think it is right to have it here (at this level of care), if the baby recovers because of CPAP, we do not need to refer him. And we do not have to spend time explaining the referral, asking for consent for the referral. We are saving time. I think it a good thing to use CPAP." (l1008) | "They (the nurses) feel happy because they see the improvement of the babies because of this intervention. They are able to use the device. They see improvements resulting from their own action." (l991) | "No, there is no negative impact" (l1006) |
| IDI 11: SR3SMC level 3 | "CPAP is not an invasive procedure, we have trained the MO, they will take care. We are comfortable with that." (l1101) | "Yes, we will be happier without these constraints (monitoring, staffing, shortages) but we have to continue (…) I will recommend it." (l1199) | "Usually we use it with surfactant and we have good results. Many patients are coming. We have better outcomes, if not we shift to mechanical ventilation. The recovery is faster, we can send the babies to mothers earlier." (l1098) "When we have the correct material, CPAP works well, staff is satisfied and parents are happy." (l1116) | "Staff are motivated, they have received training in the use of CPAP, you can ask them. They are motivated. They are the positive factor " (l1165) | "We don't have negative impacts. Sometimes we do not have prongs, the mobile x-ray is not working. These are some problems. The number of admissions is very high, the prongs' sizes we currently have are not adapted for all babies, and we have the same problem with caps (l1113)" (l1113) |
| IDI 12: SR4SNCU level 2 |  | "We would advise to start, it is a very good machine for the children." (l1303) | "*All* the babies we put under CPAP have survived." (l1247) "Parents are very happy. Previously we had to refer patients to Chennai and Hyderabad. Presently we are treating them here." (1252) | "They are doing well, the staff is very much interested in working with the CPAP." (l1251) | "No(negative impact)." (l1261) "There is no problem, we hare happy (l1320) |
| IDI 13: N2SNCU level 3 | "Then, we can use CPAP during KMC, we can continue feeding them." (l1335) "they (nurses)are happy because we use less mechanical ventilation." (l1347) "It is easy to start, but you need to be able to monitor the patients’ condition." (l1432) "It can be used everywhere provided that training and monitoring have been delivered." (l1445) | "We are still using CPAP (despite difficulties), we don’t want to lose the babies. If it becomes too difficult then we refer." (l1381) |  | "Now, they (the nurses) suggest when we have to use CPAP. I just say yes and they start. So they are more motivated." (l1365) |  |
| IDI 14: P2SNCU level 2 |  | "I will advise 100%. We can’t afford mechanical ventilation, so for mild respiratory distress CPAP will help the babies. But they (staff) need training. They need a workshop to train everyone, including nurses." (l1545) | "It helps to treat RD in babies, it provides good recovery. It reduces the need of referrals to medical colleges." (l1452) "Previously we had to refer patients, but now we have enough machines, we have 4 machines, so in general we have enough to provide care to our patients and we refer less. That is an enabling factor." (l1479) | "Since we use CPAP, we have more admissions to the SNCU and we are able to admit them with more confidence. Confidence in treating babies with mild respiratory distress and in preterm to treat recurrent apnoea. We are able to provide better treatment to low birth weight babies." (l1453) "Yes, they (the staff) are able to manage the babies, they got local training on CPAP, and babies are recovering which gives a positive feedback to their work. Whenever a baby goes home, every nurse feels happy. That is a positive impact that we have." " Moreover, middle class families who did not use to come to Government facilities, now are using our service, as reputation has improved. They do not go to private hospitals. They don’t spend money in private hospitals and they think they can receive good treatment here." (l1460) |  |
| IDI 15: PG1NICU level 3 |  | "...regarding CPAP, many secondary level hospital do not have them, only people working in tertiary units know about CPAP. I am very happy CPAP is available here, I am trained in using it and I use it. My friends in other medical colleges, they are not using CPAP. It would be better if it is more available, even in secondary level facilities and even PHC."(l1594) "(but) monitoring is essential, it will allow to assess if the baby needs to be shifted under mechanical ventilation, and also to be aware of complications like pneumothorax." (l1655) | "It reduces the need for mechanical ventilation, it helps controlling the rate of sepsis. It allows to treat preterm babies, even without surfactant, it improves the survival overall." (l1567) |  | "No we don't (have negative impact)". L1576) |
| IDI 16: MO7SNCU level 2 | "No, no increase. Workload has decreased. If there is no CPAP, we are able to provide oxygen only and we have to run to the SNCU every 5 minutes to see what is the saturation, the condition or whether the baby is taking some feeds, but if we give CPAP, at least some release is given. We do monitoring every 2 hours, we feed via OG tube, everything, our workload is reduced by using CPAP." (l1717) | "Yes it is possible (to use CPAP here). Really it is possible. In every shift, every doctor and nurse is aware about CPAP; they can do it." (l1734) "It is important using CPAP, because we are the DH, other places, small towns, do not have SNCUs, they do not have CPAP, so we receive referrals from there. They refer because they know we have CPAP. So it is really important to have CPAP here." (l1841) | "by using CPAP we are giving maximum life to babies, because most of the babies come with respiratory distress and recurrent apnoea so we give CPAP for 1-2 days, without surfactant, only CPAP. Then they recover within 2-3 days and then we use oxygen, we use mainly in preterm babies. Most of them they recover; but some babies are referred to Tirupati because they become worst." (l1685) | "Yes, we are satisfied, but we will be more satisfied if we could intubate, give surfactant and then CPAP. I think this is better than doing only CPAP. Then we are satisfied. But it is a minimal satisfaction by using only CPAP." (l1695) | "No we are not facing any difficulty, we are forming a team the three Mo and the nurses, we discuss the cases we did not get any difficulty. If we get any problem, we call the HoD and we take suggestions from him." (l1755) |
| IDI 17: MO8SNCU level 3 | "Uneasy, uneasiness, how to start with it? If we have kept around 10 to 15 (babies) under CPAP we will be more interested and more enthusiastic to keep all Respiratory distress cases under CPAP." (l1945) "We are not using it very often, so we are scared to use it, we are scared of pneumothorax or any other injury. But we don’t use it enough." (l2012) | "We want to use CPAP more. If a paediatrician is here to supervise us on how to use it, more often, then we will find it better to save lives." (l2039) | "CPAP, we use it when babies have good respiratory efforts, if we start CPAP, it will assist those respiratory efforts and we will reduce the respiratory distress better (...) (I have seen this) in a few babies, not many" (l1857) | Nothing too much to say, we are happy, a few cases have recovered. |  |
| IDI 18: SR5NICU level 3 |  | "We will (continue )using CPAP, but if we had more staff...,and sometimes babies do not get CPAP because there are not enough machines. If we can rectify this and shortage of staff we can improve quality of work (...) Definitely it has a positive impact on preterm and term babies, but still we have to improve the quality of our work." (l2184) | "A lot of babies, particularly 2-1.5 kg babies recover under CPAP only. Without needing mechanical ventilation. If surfactant is used outcome is very good." (l2046) "The incidence of apnoea decreases with early CPAP. Duration of stay in hospital decreases." (l2056) |  |  |
| FGD 1: level 3 | "We do not need doctors to operate CPAP. Doctors are very busy and mostly not available. We can operate the CPAP by ourselves. We cannot do the same with ventilators." (l21) | "If there are no CPAP machines, the babies will be put on the ventilator, which is not good, very disheartening to see babies on the ventilator. Our work load has been high since a long time. However, removing CPAP machines will not decrease the work load, instead our attention will be more focused on other babies in the SNCU. The situation will not change. Removing the CPAP is not the solution." (l398) | "We get a lot of low birth weight babies. By using CPAP, we can recover the babies, without putting them on the ventilator. We can use CPAP and observe them. If the saturations are maintained, we can wean off the babies from CPAP and connect to another oxygen supply. Else if the baby is not recovering, we can use the ventilator." (l9) "Prolonged ventilation causes sepsis and infection because of prolonged use of ET tube. So, in these cases, if the baby is weaned of during post extubation stage and given CPAP, the babies recover soon." (l67) |  |  |
| FGD2: level 2 | "We are trained to work on CPAP. So, its easy once you’ve learnt it." (l75) |  | "Earlier, when we get babies with severe respiratory distress, we used to refer them to other facilities. Now, we can manage it ourselves. The outcome is good." (l23) "But we feel through our experience, that CPAP is good for the babies. We lack facilities to support the functioning of CPAP." (l439) "We will struggle harder and face the challenges like getting supplies etc. We are coping with the issue of small staff anyway. But we see every time that CPAP is helping and it’s a relief for the babies." (l452) |  | "there are no drawbacks." (l35) |
| FGD 3: level 2 | "The machine is easy to use." (l81) |  | "Yes. It is useful. The other day, a preterm baby was brought in, with respiratory distress and heavy chest retractions. We discussed with the doctors and decided on CPAP. We explained to the parents about the condition. The parents were sad and felt they have almost lost the baby. We also explained to them the CPAP treatment, for which they gave their consent. After the treatment, the baby survived. When we told the parents, you should have seen the happiness in their face." "Another preterm baby called Arunasree was brought in with continuous apneic spells. She weighed around one kg. Even when we put her on Oxygen, we were not able to maintain the saturation levels. Then we put on CPAP. Now her spells have stopped and her saturation levels are normal." "Those days when we did not have CPAP, we used to refer preterm and low birth weight cases with apneic spells to other hospitals. Now, we handle all the cases with apnea spells or respiratory distress, or chest retraction or babies with aspiration or pneumonia and we are able to make good progress." (l8) "We are able to see good changes in babies who are brought in with respiratory distress or apneic spells. Earlier, we used to refer them to other facilities. Now, with CPAP, we are able to manage things on our own. If the problem is identified fast and CPAP is administered soon, I think there will be more chance of recovery." (l47) | "Now that we are trained, we are confident and can operate independently, without the doctors. We were trained by Dr.S.Murthy who took a session on what the machine is all about, parts of the machine, how to connect the machine, benefits of the machine, on which babies should it be administered and what is the nursing care that should be given during that time, how to calculate the Silverman’s score. We are very happy that we can do things independently." (l27) |  |
| FGD 4: level 3 |  |  | "It has reduced the respiratory distress. Recurrent apnea has been reduced. Babies have been saved. It is very useful for preterm babies, 70%. After the post extubation phase, we have been able to save the babies through CPAP, esp. the babies with very low birth weight." (l23) "We have saved babies weighing even less that one kg, on CPAP." (l54) "Yes. It is useful. Respiratory distress in babies is reduced. We are using it on preterm babies and saving them. If we had more machines, it will be helpful." (l74) | "We, nurses, operate the machines on our own, many times without the help of doctors- be it identifying the baby to be administered on CPAP or placing the prongs, positioning the baby, or recording the Silverman score- we do it by ourselves." (l59) |  |
| FGD 5: level2 |  |  |  | "There was a situation when there were two of us in the ward and we had a baby brought in with severe grunting and respiratory distress. The doctor was not available. So we told him the situation on telephone. He said if you think CPAP is needed, go ahead. We connected the CPAP. Luckily, the baby’s condition improved the next day and the doctor came and appreciated us." (l101) "No. in fact, we are very confident to connect to CPAP. We began taking cases even when the doctors are not available. We call them on phone and explain the condition, vitals , saturations etc. When they ask us to give CPAP, we connect the machine." (l133) | "We did not encounter any problems using our machine." (l11) |
| FGD 6: level 3 | "It is easy to place easy to remove, it is not difficult to manage CPAP." (l35) |  | "Putting the baby on ventilator leads to more injuries, air way injuries. So through CPAP, injuries are less so CPAP machine is useful. Much more useful for preterm babies with respiratory distress, grunting, retraction,..., post intubation babies, ah and recurrent apneas without medical management. For this type of patients, it is very useful." (l22) "we can prevent recurrent apneas also, recurrent apneas normally occurred during oxygen supply but with CPAP we can prevent recurrent apneas also." (l54) "here we are used to get more preterm babies so CPAP is useful." (l59) |  |  |
| FGD 7: level 2 | "We have no problem in connecting the CPAP machine and we are well trained and are able to operate the machine with ease since the first demonstration itself." (l61) | There are more advantages than disadvantage of using CPAP (l57) | "Yes. It is useful for term babies with distress and they recover by the third day on CPAP support." (l39) "Otherwise CPAP is saving most of the babies from being intubated. There are more advantages than disadvantages of CPAP. Babies have recovered in two or three days. We have weaned off the some of the babies even in few hours when they have shown signs of recovery." (l57) "Even referrals have come down because of CPAP. Earlier, when we see (respiratory) distress getting higher, we refered for ventilation to another hospital." (l151) "Babies with meconium and those born preterm have very low chances of survival, even with CPAP." (l40) | "The doctors are not available from 2 in the afternoon to 8 early morning. The nurses manage everything. Most of their skills improved because they had to do things on their own." (l96) "We are now used to it. We are also used to working independently, without doctors. We had struggled one month without doctors. They used to come only when they are called. Sometimes. When emergency strikes, we have to discuss on phone." (l269) |  |
| FGD 8: level 3 |  |  | "CPAP is useful to control respiratory distress in babies and the change is more visible in term babies than preterm babies. CPAP machines are useful when you are weaning the babies from the ventilator, It helps to treat premature babies with apneic attacks. These babies recover soon with CPAP." (l15) "Mainly CPAP does not work on preterm babies because saturation levels go low and we have to connect the endotracheal tube." (l53) "If preterm babies are given surfactant injection before connecting to CPAP, respiratory distress can be controlled." (l58) | "The doctors check the vitals and after observation, advise CPAP when necessary. We, nurses then, make the preparations for CPAP and connect the baby. If the doctors are busy attending to gynaec cases and are not available then, we decide on CPAP if the baby is grunting excessive and has severe respiratory distress." (l157) |  |
